# Anticancer Potential of Damnacanthal and Nordamnacanthal from *Morinda elliptica* Roots on T-lymphoblastic Leukemia Cells

**DOI:** 10.3390/molecules26061554

**Published:** 2021-03-12

**Authors:** Saiful Yazan Latifah, Banulata Gopalsamy, Raha Abdul Rahim, Abdul Manaf Ali, Nordin Haji Lajis

**Affiliations:** 1 Department of Biomedical Sciences, Faculty of Medicine and Health Sciences, Universiti Putra Malaysia, Serdang 43400 UPM, Selangor, Malaysia; banulata@upm.edu.my; 2 Department of Cell and Molecular Biology, Faculty of Biotechnology and Biomolecular Sciences, Universiti Putra Malaysia, Serdang 43400 UPM, Selangor, Malaysia; raha@upm.edu.my; 3 Faculty of Bioresources and Food Industry, Universiti Sultan Zainal Abidin (UniSZA), Kuala 20300, Terengganu, Malaysia; manaf@unisza.edu.my; 4 Laboratory of Natural Products, Institute of Bioscience, Universiti Putra Malaysia, Serdang 43400 UPM, Selangor, Malaysia; nordinlajis@gmail.com

**Keywords:** damnacanthal, nordamnacanthal, anticancer, CEM-SS, apoptosis, Mg^2+^/Ca^2+^-dependent endonuclease, G0/G1 arrest, cytotoxic

## Abstract

Background: This study reports on the cytotoxic properties of nordamnacanthal and damnacanthal, isolated from roots of *Morinda elliptica* on T-lymphoblastic leukaemia (CEM-SS) cell lines. Methods: MTT assay, DNA fragmentation, ELISA and cell cycle analysis were carried out. Results: Nordamnacanthal and damnacanthal at IC_50_ values of 1.7 μg/mL and10 μg/mL, respectively. At the molecular level, these compounds caused internucleosomal DNA cleavage producing multiple 180–200 bp fragments that are visible as a “ladder” on the agarose gel. This was due to the activation of the Mg^2+^/Ca^2+^-dependent endonuclease. The induction of apoptosis by nordamnacanthal was different from the one induced by damnacanthal, in a way that it occurs independently of ongoing transcription process. Nevertheless, in both cases, the process of dephosphorylation of protein phosphates 1 and 2A, the ongoing protein synthesis and the elevations of the cytosolic Ca^2+^ concentration were not needed for apoptosis to take place. Nordamnacanthal was found to have a cytotoxic effect by inducing apoptosis, while damnacanthal caused arrest at the G0/G1 phase of the cell cycle. Conclusion: Damnacanthal and nordamnacanthal have anticancer properties, and could act as potential treatment for T-lymphoblastic leukemia.

## 1. Introduction

Leukaemia, a malignant disorder involving the bone marrow and blood cells, is a life-threatening health problem. Leukaemia recorded 437,033 new cases and 309,006 leukaemia-related death in the year 2018. This number accounts for 2.4% of all types of cancer and 3.2% of the total cancer-related deaths [1,2]. Of the recent statistics, leukaemia is the 14th-most-recorded type of cancer, and the 11th-highest death-causing cancer [1]. Lymphoblastic leukaemia occurs in both adults and children, but almost 60% of cases are reported to be in those younger than 20 years of age. This disorder exists with biologically distinct subtypes, making it difficult to comprehend the causal mechanism. Its cause is multifactorial, involving genetic susceptibility, exposure to endogenous and exogenous causative agents and also chance [3].

Since lymphoblastic leukaemia is not concentrated to a specific site, systemic chemotherapy is the main treatment modality. Radiation, immunotherapy and targeted therapy and stem cell transplantation combinations therapy are among the other forms of treatment for this disease [4]. This form of cancer, if well managed, has a very high chance of cure, whereby developed countries with access to top chemotherapy and recent protocols record more than an 80–90% survival rate. The five-year survival rate in the Asian region is 44.3–80%, and upper-middle and high-income countries reported five-year event-free survival rates of 74% [5]. Since relapses still occur, newer treatments of drugs to treat this condition are warranted.

**Figure 1:**
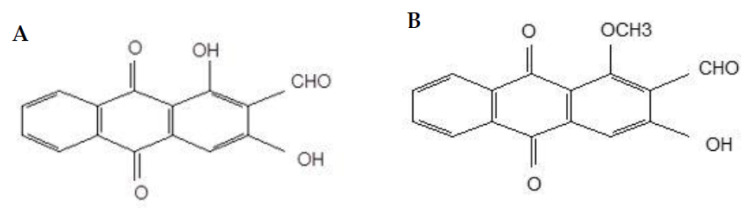
Chemical structure of nordamnacanthal (**A**) and damnacanthal (**B**).

*Morinda elliptica* of the Rubiaceae family has been documented since early times for its vast medicinal properties. *M. elliptica* is commonly recognised as mengkudu kecil in the Malay language. Burkil [6] reported the traditional use of this plant species to treat wounds, fever, diarrhea, cholera, poor appetite, headache and other minor discomforts by locals. The extract of *M. elliptica* exhibit profound antioxidant, antitumor [7], antimicrobial, antiviral, cytotoxic [8], antileukemic and anticancer properties. Two naturally occurring anthraquinones successfully isolated from *M. elliptica* are nordamnacanthal (Figure 1A) and damnacanthal (Figure 1B).

Nordamnacanthal and damnacanthal have profound health benefits. Even though many constituents in *M. elliptica* active extract were identified and studied, damnacanthal and nordamnacanthal are reported to exhibit a wide range of medicinal properties, specifically in cancer. These anthraquinones are cytotoxic against the human breast cancer cell line, MCF-7 and MDA-MB231, and myelogenous leukemia cell line, K-562 [9,10,11]. Damnacanthal specifically exhibits antitumorigenic activity in human colorectal cancer cells, H1299 and HCT-116, by inducing caspase activity and cell growth arrest. Furthermore, damnacanthal enhances transcription factor CCAAT/enhancer binding protein β (C/EBPβ), which subsequently augments the transcription of proapoptotic protein nonsteroidal anti-inflammatory activated gene-1 (NAG-1) [12,13]. Shaghayegh et al. [14] demonstrated H400 oral squamous cell carcinoma undergoing apoptotic morphological alterations, the inhibition of cell proliferation and cell migration, as well as early apoptosis induction upon treatment, with both damnacanthal and nordamnacanthal suggesting it to be ideal for oral cancer therapy. 

Damnacanthal, present in different parts of *M. citrifolia*, including roots and fruit peel, inhibits the tyrosine kinases, which include c-Methionine, in exhibiting chemo preventive properties in human hepatocellular carcinoma Hep G2 [15]. In vivo treatment with nordamnacanthal effectively reduced 4T1 tumor sizes in murine cancer cells-challenged mice, and amplifies T helper, NK and cytotoxic T cells’ population [11]. They were also able to rule out the possible toxic effect of this anthraquinone in the mice model, indicating that this compound is safe to be used. These anthraquinones also pose antioxidant activities, free radical scavenging and antitumor activities, making them ideal for the treatment of cancer [7,16,17]. 

The broad spectrum of bioactivities displayed by damnacanthal and nordamnacanthal suggest their potential for clinical application, specifically as anticancer agents. Since consistent desired outcomes were observed in an array of cancer cell lines upon treatment with these anthraquinones, they could potentially be a treatment for lymphoblastic leukaemia. Therefore, in this study we investigated the cytotoxic effect of damanacanthal and nordamnacanthal treatment in T-lymphoblastic leukaemia (CEM-SS) cells. We also report the possible involvement of the downstream mechanisms of damnacanthal and nordamnacanthal in exhibiting its anticancer properties. 

## 2. Results

### 2.1. Cytotoxicity of Nordamnacanthal and Damnacanthal

**Figure 2:**
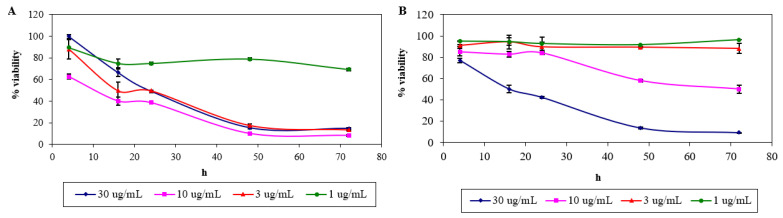
The percentage of viability (relative to control) of T-lymphoblastic leukaemia (CEM-SS) cells treated with different concentrations of nordamnacanthal (**A**) and damnacanthal (**B**) for a 72 h period, determined using the MTT assay. Control cultures were not treated with nordamnacanthal or damnacanthal. Each data point represents the mean of three independent experiments, and vertical lines through the data points indicate standard deviation.

The MTT assay showed a reduction in the cell viability by time, with apparent different lower percentage in every concentration of the respective compounds (relative to untreated control) used in the experiment. At 24 h, the fall in cell viability of treatment using 30, 10, 3 and 1 μg/mL of nordamnacanthal were 49%, 39%, 50% and 75%, which further declined to 15%, 8%, 14% and 69% at 72 h, respectively (Figure 2A). The IC_50_ value of nordamnacanthal is determined to be 1.7 μg/mL.

In the case of damnacanthal, a marked drop in cell viability at 24 h (50%) was exhibited, but only at the highest concentration of 30 μg/mL of the compound, which went down to 9% at 72 h. Fifty percent reduction in the cell viability was exhibited at 72 h by the treatment of 10 μg/mL of the compound. On the other hand, the percentage of cell viability remained high in the treatment of lower concentrations of damnacanthal, 3 and 1 μg/mL, i.e., 88% and 96%, respectively (Figure 2B). The IC_50_ value of damnacanthal is calculated to be 10 μg/mL.

### 2.2. Effects of Nordamnacanthal and Damnacanthal on DNA Fragmentation in CEM-SS Cells

Both nordamnacanthal and damnacanthal caused DNA fragmentation in CEM-SS cells, producing fragments of multiples of 200 base pairs that appeared as a distinctive ladder-like pattern on agarose gel, the most prominent biochemical hallmark of apoptosis. 

**Figure 3:**
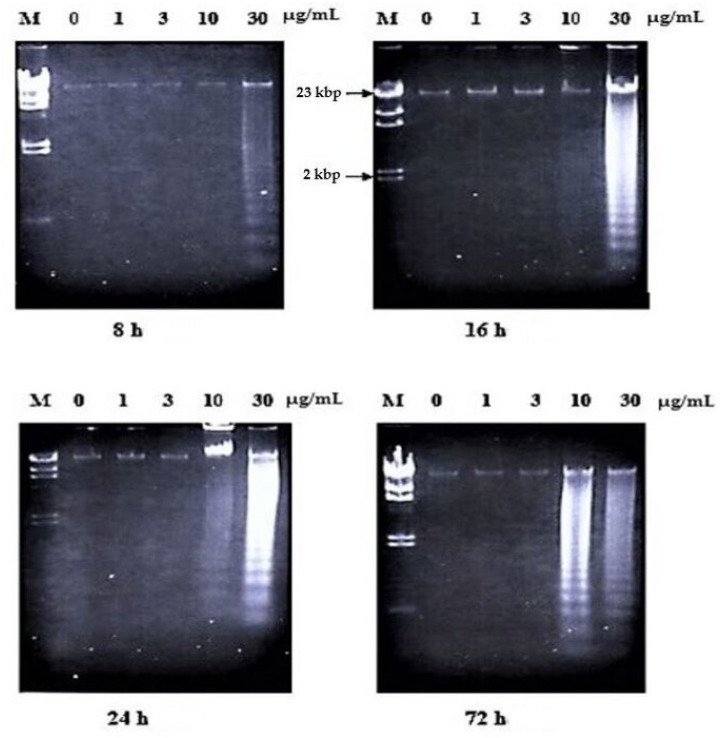
Effects of different concentrations of nordamnacanthal at different hours on DNA fragmentation in CEM-SS cells. Lane M: Marker (HindIII digest of lambda DNA).

In the case of nordamnacanthal, ‘smearing’ could be observed as early as after 6 h (data not shown), followed by an extremely clear ‘ladders’ formation after 8 h, which remained until 72 h using 30 μg/mL of the respective compound. In the treatment, using 10 μg/mL of nordamnacanthal, the DNA fragmentation started with slight degradation of DNA after 16 h, and the ‘ladders’ could only be clearly seen after 24 h. However, there were no ‘ladders’ that could be detected in the treatment of 3 and 1 μg/mL of the compound (Figure 3).

**Figure 4:**
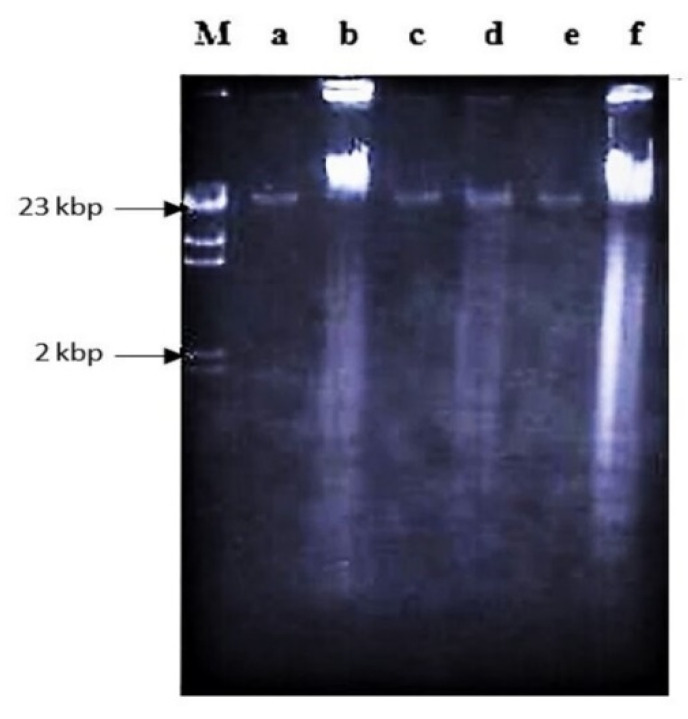
The involvement of Ca^2+^/Mg^2+^-dependent endonuclease and protein synthesis in nordamnacanthal-induced apoptosis in CEM-SS cells at 24 h. Zinc sulphate and cycloheximide failed to prevent apoptosis induced by 10 µg/mL of nordamnacanthal at 24 h. Lane a: control. Lane b: nordamnacanthal (10 µg/mL). Lane c: zinc sulphate (0.1 mM). Lane d: nordamnacanthal (10 µg/mL) + zinc sulphate (0.1 mM). Lane e: cycloheximide (10 µg/mL). Lane f: nordamnacanthal (10 µg/mL) + cycloheximide (10 µg/mL). Lane M: Marker (HindIII digest of lambda DNA).

**Figure 5:**
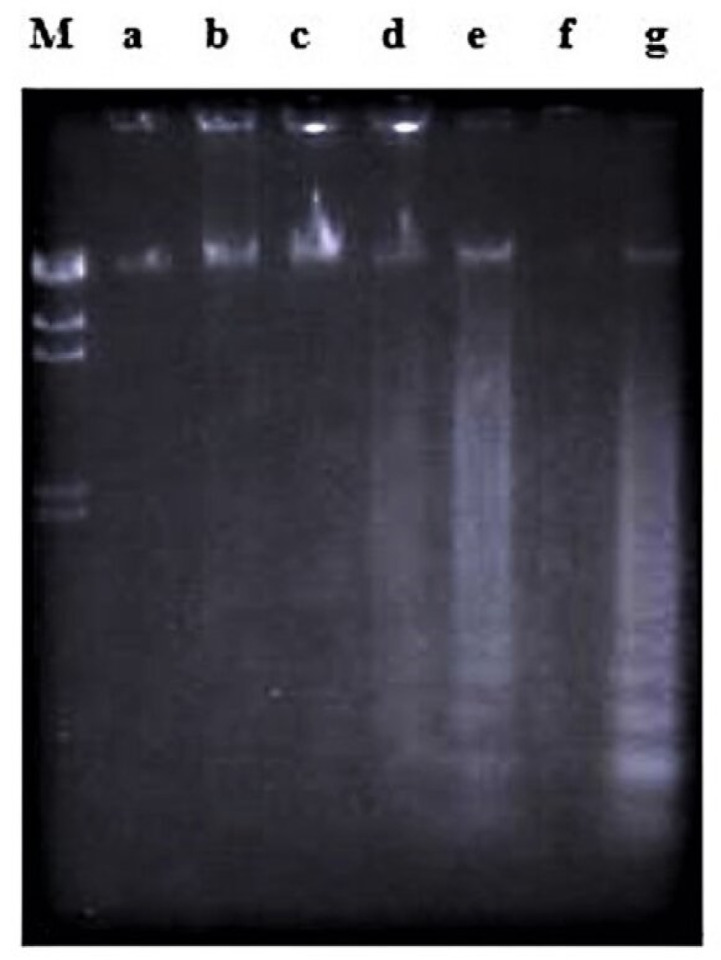
The involvement of RNA synthesis in nordamnacanthal-induced apoptosis in CEM-SS cells at 6 and 8 h. Actinomycin D failed to prevent apoptosis induced by 30 µg/mL of nordamnacanthal at 6 and 8 h. Lane a: control. Lane b: nordamnacanthal (30 µg/mL) (6 h-treatment). Lane c: actinomycin D (1 µg/mL) (6 h-treatment). Lane d: nordamnacanthal (30 µg/mL) + actinomycin D (1 µg/mL) (6 h-treatment). Lane e: nordamnacanthal (30 µg/mL) (8 h treatment). Lane f: actinomycin D (1 µg/mL) (8 h-treatment). Lane g: nordamnacanthal (30 µg/mL) + actinomycin D (1µg/mL) (8 h-treatment). Lane M: Marker (HindIII digest of lambda DNA).

**Figure 6:**
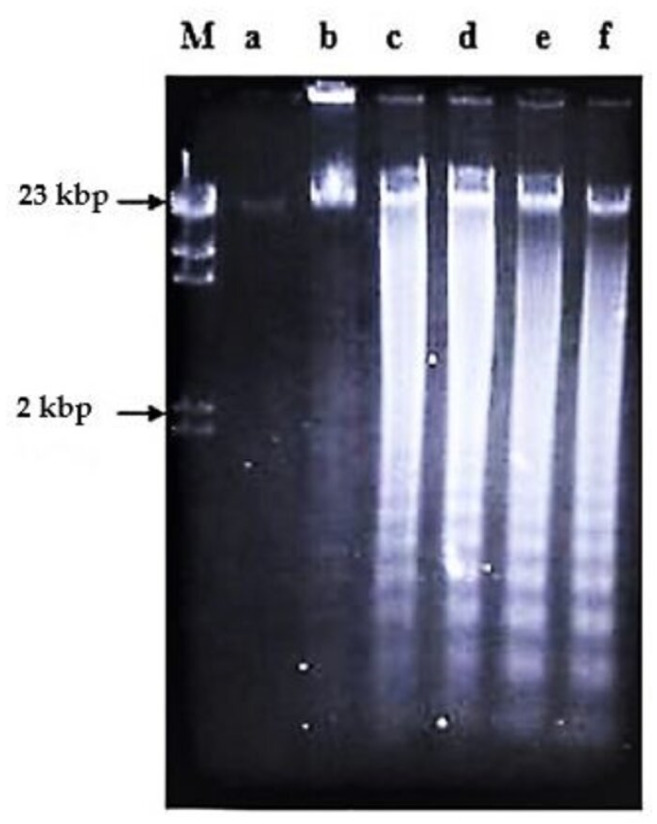
The involvement of phosphatases and RNA synthesis in nordamnacanthal-induced apoptosis in CEM-SS cells at 24 h. Okadaic acid and actinomycin D failed to prevent apoptosis induced by 10 µg/mL of nordamnacanthal at 24 h. Lane a: control. Lane b: nordamnacanthal (10 µg/mL). Lane c: okadaic acid (100 nM). Lane d: nordamnacanthal (10 µg/mL) + okadaic acid (100 nM). Lane e: actinomycin D (1 µg/mL). Lane f: nordamnacanthal (10 µg/mL) + actinomycin D (1 µg/mL). Lane M: Marker (HindIII digest of lambda DNA).

**Figure 7:**
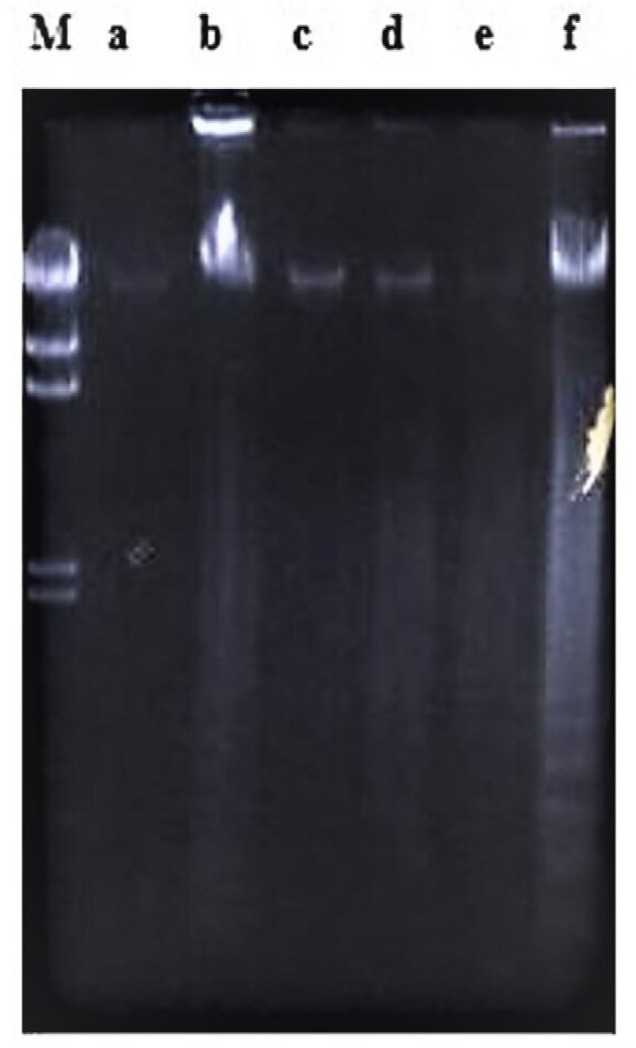
The involvement of an increase in cytosolic calcium concentration in nordamnacanthal-induced apoptosis in CEM-SS cells at 24 h. EGTA failed to prevent apoptosis induced by 10 µg/mL of nordamnacanthal at 24 h. Lane a: control. Lane b: nordamnacanthal (10 µg/mL). Lane c: EGTA (1 mM). Lane d: nordamnacanthal (10 µg/mL) + EGTA (1 mM). Lane e: EGTA (8 mM). Lane f: nordamnacanthal (10 µg/mL) + EGTA (8 mM). Lane M: Marker (HindIII digest of lambda DNA).

Treatment using a combination of nordamnacanthal with a concentration known to induce apoptosis (10 μg/mL) and zinc sulphate (0.1 mM) or cycloheximide (10 μg/mL) (Figure 4), actinomycin D (1 μg/mL) (Figure 5 and Figure 6), okadaic acid (100 nM) (Figure 6) or EGTA (1 and 8 mM) (Figure 7) for 24 h failed to prevent the existence of the ‘ladder’. In fact, in the treatment employing okadaic acid (100 nM), actinomycin D (1 μg/mL) and EGTA (8 mM) alone, the ‘ladders’ occurred. Even though actinomycin D at 1 μg/mL itself did not produce DNA fragments after 6 and 8 h of treatment, it was still incapable of inhibiting not only the ‘ladder’ formation, but also the ‘smearing’ caused by 30 μg/mL of nordamnacanthal, respectively (Figure 5).

**Figure 8:**
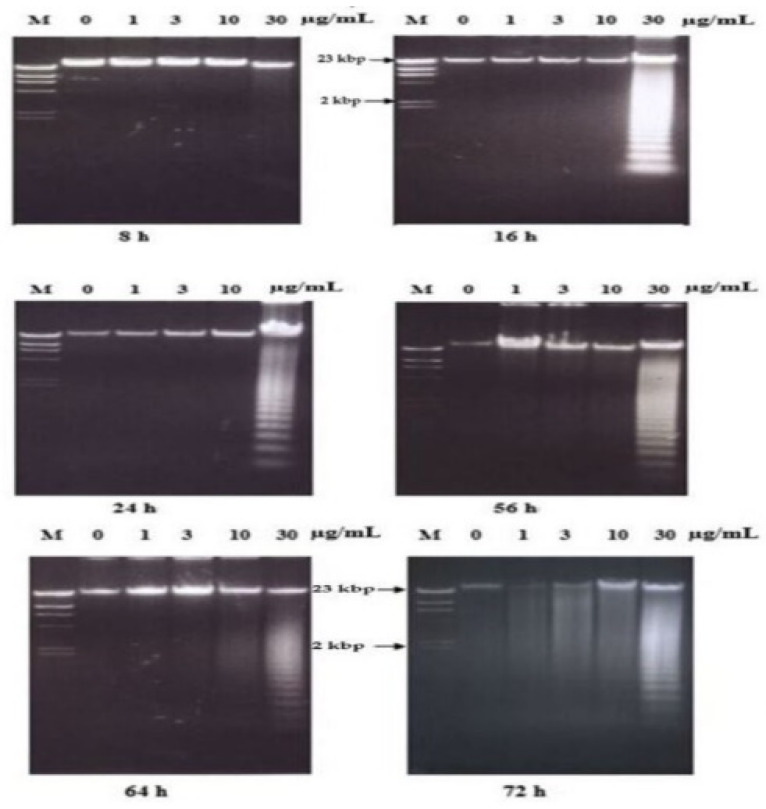
Effects of different concentrations of damnacanthal at different hours on DNA fragmentation in CEM-SS cells. Lane M: Marker (*Hind*III digest of lambda DNA).

**Figure 9:**
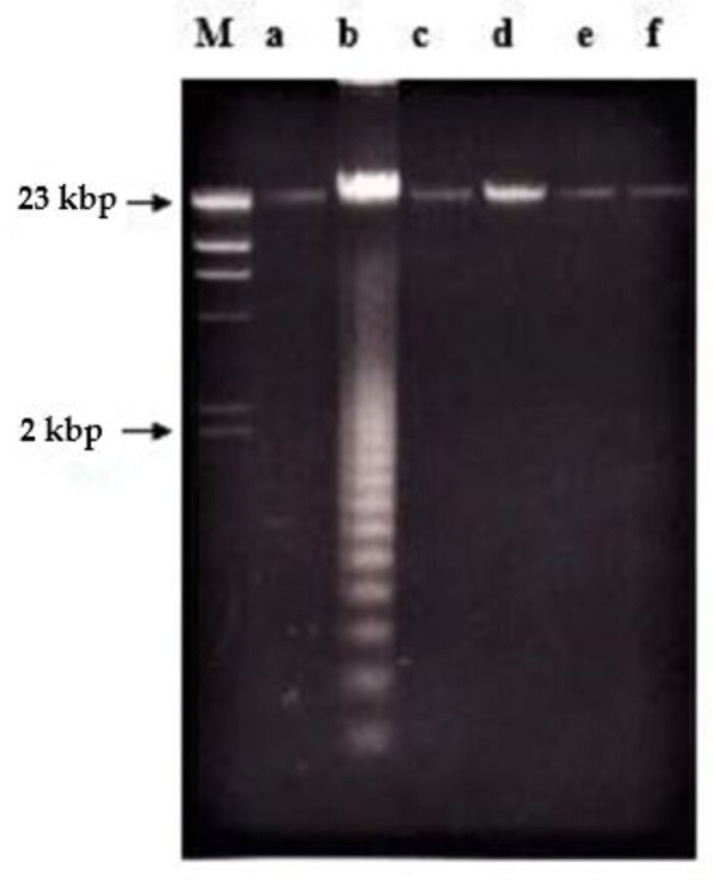
The involvement of Ca^2+^/Mg^2+^-dependent endonuclease and protein synthesis in damnacanthal-induced apoptosis in CEM-SS cells at 24 h. Zinc sulphate and cycloheximide prevented apoptosis induced by 30 µg/mL of damnacanthal at 24 h. Lane a: control. Lane b: damnacanthal (30 µg/mL). Lane c: zinc sulphate (0.1 mM). Lane d: damnacanthal (30 µg/mL) + zinc sulphate (0.1 mM). Lane e: cycloheximide (10 µg/mL). Lane f: damnacanthal (30 µg/mL) + cycloheximide (10 µg/mL). Lane M: Marker (HindIII digest of lambda DNA).

**Figure 10:**
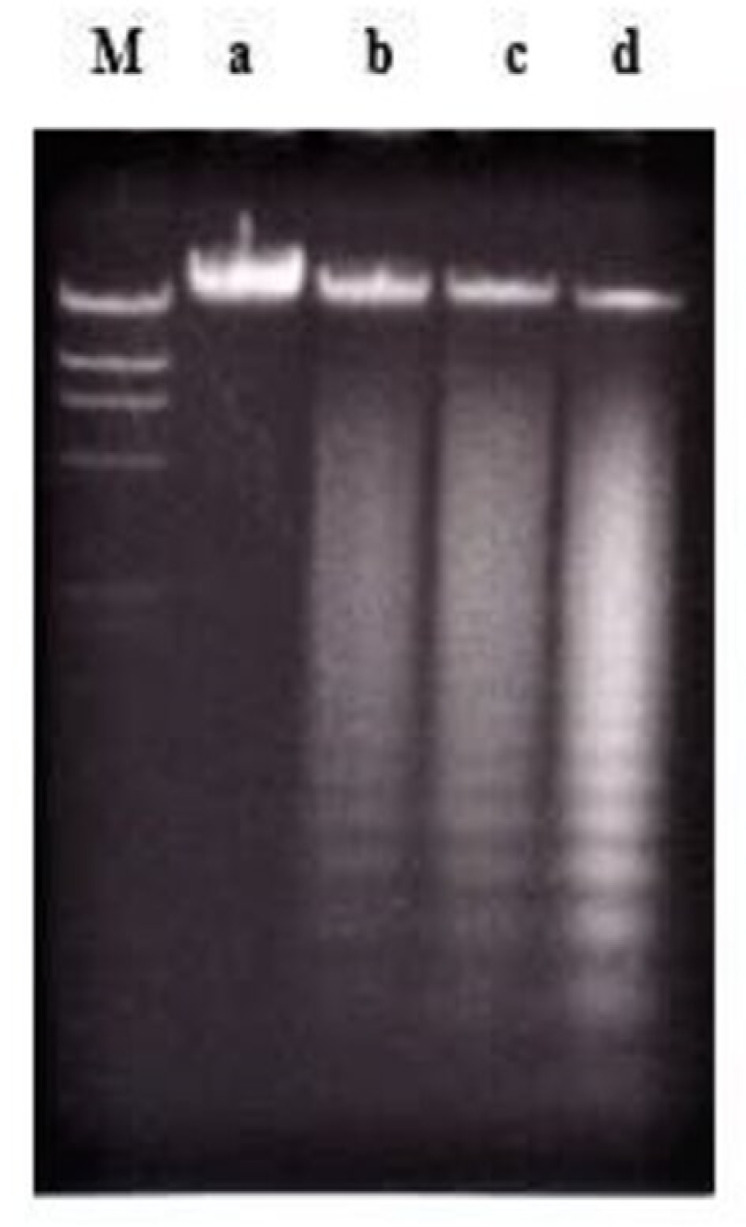
The involvement of RNA synthesis in damnacanthal-induced apoptosis in CEM-SS cells at 24 h. Actinomycin D failed to prevent apoptosis induced by 30 µg/mL of damnacanthal at 24 h. Lane a: control. Lane b: damnacanthal (30 µg/mL). Lane c: actinomycin D (1 µg/mL). Lane d: damnacanthal (30 µg/mL) + actinomycin D (1 µg/mL). Lane M: Marker (HindIII digest of lambda DNA).

**Figure 11:**
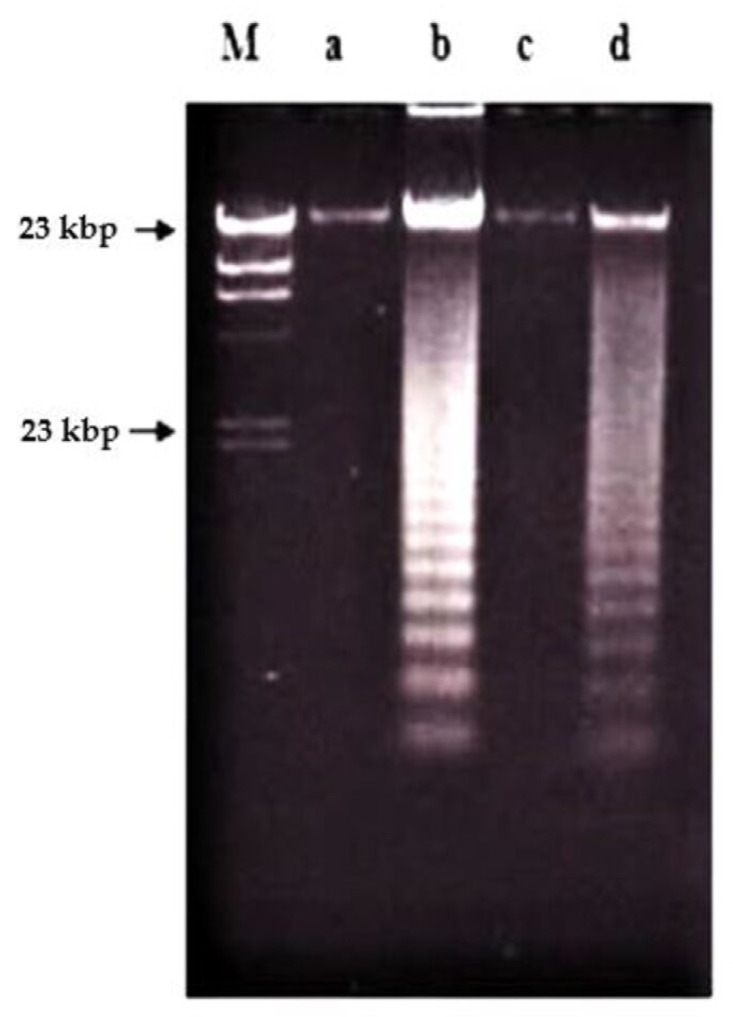
The involvement of RNA synthesis in damnacanthal-induced apoptosis in CEM-SS cells at 24 h. Actinomycin D failed to prevent apoptosis induced by 30 µg/mL of damnacanthal at 24 h. Lane a: control. Lane b: damnacanthal (30 µg/mL). Lane c: actinomycin D (10 µg/mL). Lane d: damnacanthal (30 µg/mL) + actinomycin D (10 µg/mL). Lane M: Marker (HindIII digest of lambda DNA).

**Figure 12:**
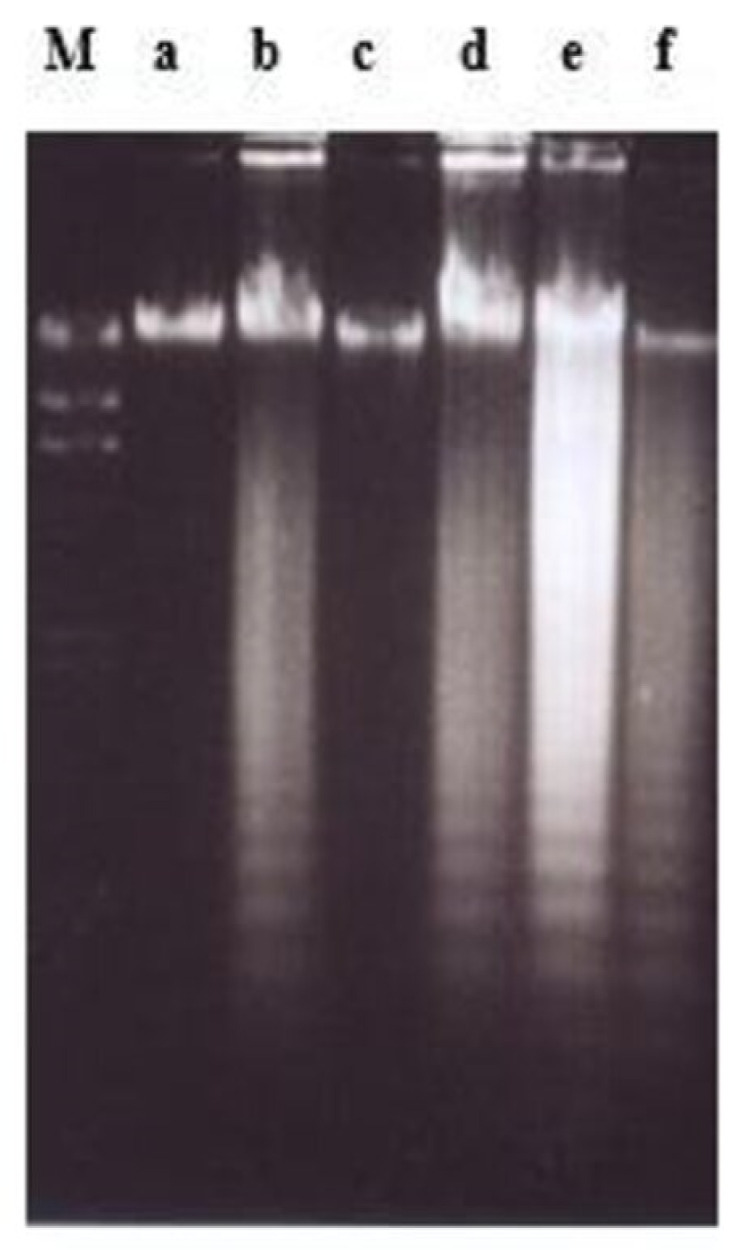
The involvement of increase in cytosolic calcium concentration and phosphatases in damnacanthal-induced apoptosis in CEM-SS cells at 24 h. EGTA and okadaic acid failed to prevent apoptosis induced by 30 µg/mL of damnacanthal at 24 h. Lane a: control. Lane b: damnacanthal (30 µg/mL). Lane c: EGTA (0.5 mM). Lane d: damnacanthal (30 µg/mL) + EGTA (0.5 mM). Lane e: okadaic acid (100 nM). Lane f: damnacanthal (30 µg/mL) + okadaic acid (100 nM). Lane M: Marker (HindIII digest of lambda DNA).

**Figure 13:**
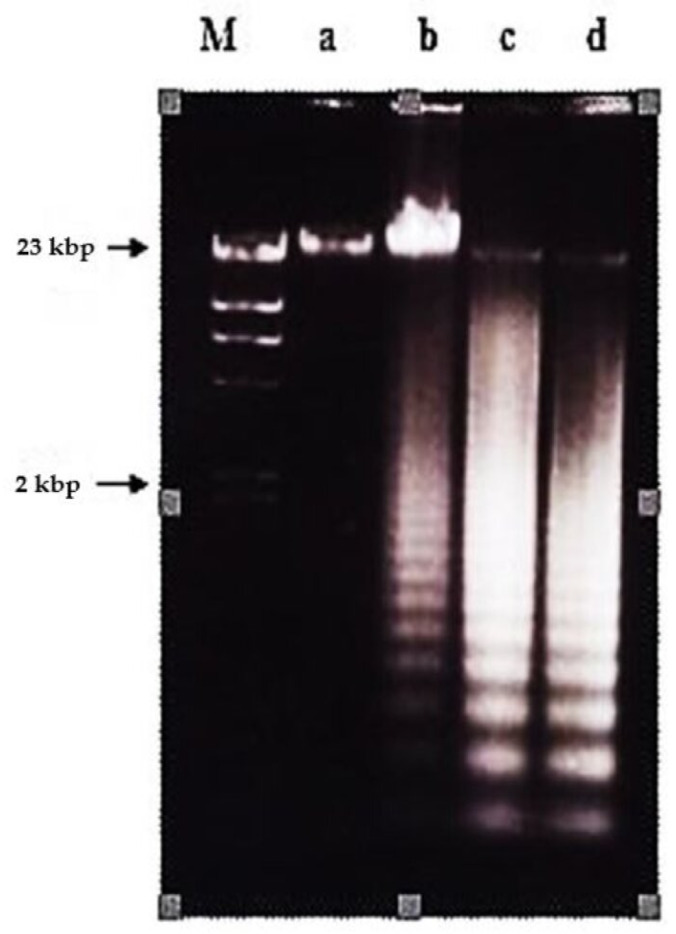
The involvement of increase in cytosolic calcium concentration in damnacanthal-induced apoptosis in CEM-SS cells at 24 h. EGTA failed to prevent apoptosis induced by 30 µg/mL of damnacanthal at 24 h. Lane a: control. Lane b: damnacanthal (30 µg/mL). Lane c: EGTA (1mM). Lane d: damnacanthal (30 µg/mL) + EGTA (1mM). Lane M: Marker (HindIII digest of lambda DNA).

Treatment using damnacanthal at 30 μg/mL caused slight DNA degradation after the first 8 h, and the ‘ladders’ can be clearly noticeable after 16 h. On the other hand, in the treatment employing another three concentrations, 10, 3 and 1 μg/mL, slight DNA degradation existed only after 56 h, and yet until 72 h, the ‘ladders’ were unable to be clearly seen (Figure 8). Unlike nordamnacanthal, the addition of zinc sulphate (0.1 mM) or cycloheximide (10 μg/mL) into the treatment using 30 μg/mL of damnacanthal for 24 h, the ‘ladders’ totally vanished (Figure 9). However, a combination of 30 μg/mL of damnacanthal and actinomycin D (1 and 10 μg/mL) (Figure 10 and Figure 11) or okadaic acid (100 nM) (Figure 12) or EGTA (0.5 and 1 mM) (Figure 12 and Figure 13) failed to stop DNA fragmentation. DNA of the untreated cells as a negative control remained intact for the whole series of the experiment.

#### 2.2.1. ELISSA

Cell Death Detection ELISAPLUS allows the specific determination of mono- and oligonucleosomes in the cytoplasmic fraction of cell lysates, which further confirmed the induction of apoptosis by nordamnacanthal and damnacanthal. The time- and dose-dependent presence of these mono- and oligonucleosomes was depicted by an increment in the absorbance (optical density) reading.

**Figure 14:**
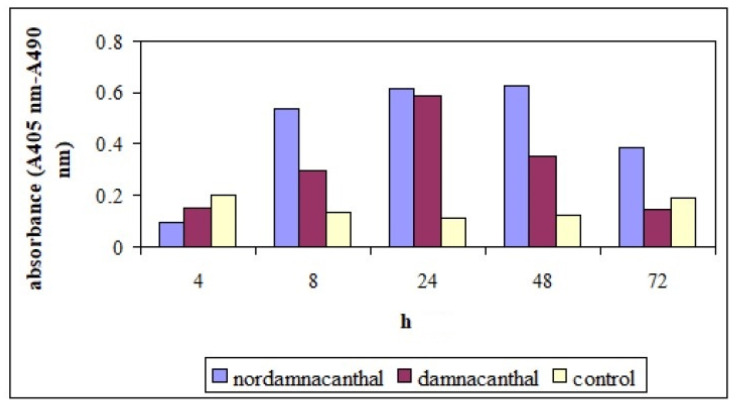
Detection of nucleosomes in cytoplasmic fractions of cell lysates at different hours of experiments. CEM-SS cells were treated with 30 μg/mL of indicated compounds. Control cultures were not treated with nordamnacanthal or damnacanthal. 20 μL of cell lysates were analyzed in the ELISA.

**Figure 15:**
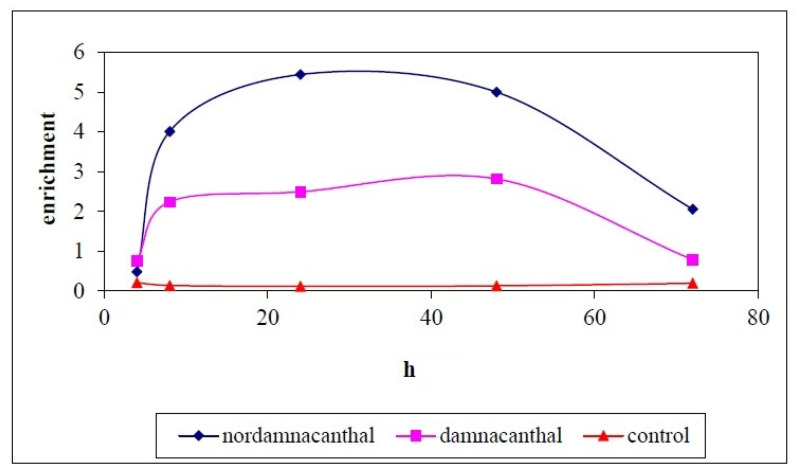
Enrichment of nucleosomes in cytoplasmic fractions of cell lysates at different hours of experiments. CEM-SS cells were treated with 30 μg/mL of indicated compounds. Control cultures were not treated with nordamnacanthal or damnacanthal. 20 μL of cell lysates were analyzed in the ELISA.

In the whole experiment, nordamnacanthal showed the highest absorbance reading compared to damnacanthal and the control, even though the reading decreased slightly after 72 h (Figure 14). In the supernatant, the level of the oligonucleosomes produced by treatment with nordamnacanthal, damnacanthal and the control was almost similar. The absorbance reading given by the control was due to the normal cell culture condition, whereby each exponentially growing permanent cell culture will contain a certain number of dead cells (normally approximately 3–8%). The higher enrichment factor in the treatment employing nordamnacanthal than damnacanthal explained the greater level of DNA fragmentation in the former (Figure 15).

#### 2.2.2. Cell Cycle Analysis

**Figure 16:**
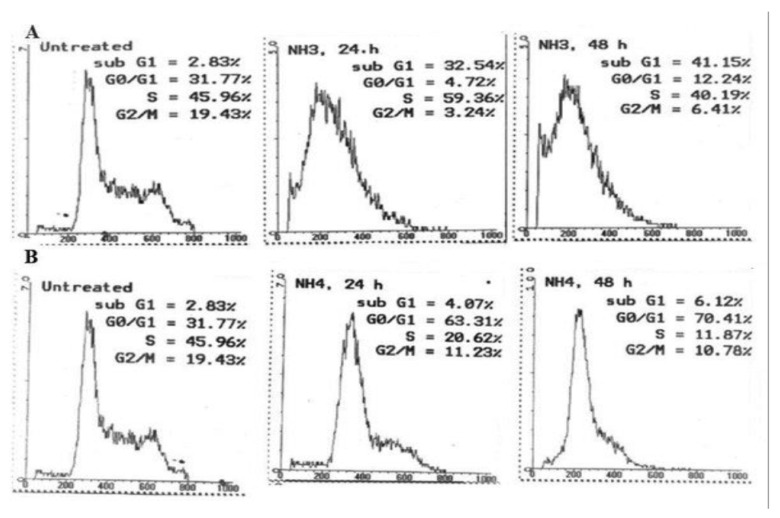
Cell cycle distribution of CEM-SS cells after 24 h and 48 h incubation with (**A**) nordamnacanthal and (**B**) damnacanthal at their respective IC_50_ values.

The changes in the cell cycle distribution of CEM-SS cells after 24 and 48 h incubation with nordamnacanthal and damnacanthal at their respective IC_50_ value are illustrated in Figure 16A,B, and summarized in Table 1. Analysis of the cell cycle profiles showed an increase in S cell population, as well as in the percentage of sub-G1 cells after 24 h of treatment with nordamnacanthal. After 48 h, however, longer exposure times led to an additional increase in the percentage of sub-G1 cells up to 41.15%. At the same time, the arrest at the S phase has been alleviated, allowing the cells to proceed to the G2/M phase.

Damnacanthal showed a steady G0/G1 arrest throughout the experiment with an increase in the percentage of cells in this phase from 63.31% to 70.41%, 24 h and 48 h after treatment, respectively. Nevertheless, the percentage of sub-G1 cells was considerably low throughout the treatment duration. 

## 3. Discussion

The IC_50_ values of nordamnacanthal and damnacanthal were 1.7 and 10 μg/mL, respectively. As they exhibited cytotoxicity at concentrations 10 μg/mL and lower, they are considered promising cancer chemotherapeutic agents. The cytotoxic effects of anthraquinones against cancerous cells have been exhibited previously. Anthraquinones from *Morinda citrifolia*, for example, were toxic against human heptacellular carcinoma HepG2 [15], oral squamous cell carcinoma [14], colorectal cancer, HT1299 and HCT-116 [12,13] and myelogenous leukemia [9].

Even though the chemical structure between nordamnacanthal and damnacanthal is closely related, except for the occurrence of methoxyl instead of methyl group at C-1 in the latter compound, there was a significant difference in their cytotoxicity towards CEM-SS cells, with nordamnacanthal shown to be more active. The existence of this different group was predicted to be the contributing factor to the decrement of the activity of damnacanthal. The number of hydroxyl groups (OH) seemed to play an important role in the degree of the cell growth inhibition by quinones on cultured malignant cells, whereby the anthraquinones with more hydroxyl groups were more effective than those with less or without the existence of the groups [18]. The position, structure and function of hydroxyl groups have major influence on the anticancer activities of an anthraquinone [19,20].

Nordamnacanthal and damnacanthal from *Morinda elliptica* exhibited another most important biochemical feature of apoptosis, besides chromatin condensation, i.e., the cleavage of the chromatin at the internucleosomal regions generating fragments that were multiples of 200 bp, visualized by DNA gel electrophoresis as a distinctive ladder-like pattern [21]. The presence of mono- and oligonucleosomes in the cytoplasmic fraction of cell lysate further confirmed the mode of cell death induced by these two compounds.

The occurrence of the ladder in this study was time- and concentration-dependent for nordamnacanthal and damnacanthal. The intensity of the ‘laddering’ pattern became stronger at 72 h in treatment using higher concentrations of the compounds (30 and 10 μg/mL), which coincided with the occurrence of secondary necrosis [21,22]. Apoptosis peaks in CEM-SS cultures treated with nordamnacanthal and damnacanthal were between 24 h and 48 h, and decreased thereafter, as the apoptotic bodies undergo secondary necrosis and were degraded to debris. Nevertheless, the ‘ladder’ pattern resulting from this internucleosomal cleavage by nordamnacanthal and damnacanthal can still be observed in DNA extracted from cultures up to three days after treatment, long after the apoptotic bodies have degenerated into featureless masses of cell debris, similar to Shaghayegh et al. [23].

At lower concentrations of 1 and 3 μg/mL, the DNA ladder existed only in the treatment using damnacanthal. Perhaps low concentrations of nordamnacanthal took a longer time for the degradation of large fragments of DNA to smallest fragments of approximately 200 bp; this was because DNA fragmentation during apoptosis proceeds through an ordered series of stages, beginning with the production of DNA fragments of 300 kbp, which are then degraded to fragments of 50 kbp. Fragments of this size are further degraded to smaller fragments of 10 to 40 kbp, and finally to small oligonucleosome fragments of 180 to 200 bp that are recognized as the characteristic DNA ladder. These phenomena could be further confirmed using pulsed-field gel electrophoresis (PGFE), which can detect the initial stages of DNA fragmentation to fragments of 300 and 50 kbp [24]. Even though nordamnacanthal and damnacanthal did induce necrosis in CEM-SS cells in the latter stage of the experiment, the smear of DNA rather than a ladder as a result of non-specific destruction of chromatin was undetectable by agarose gel electrophoresis. The high levels of apoptosis could possibly mask the occurrence of the smear.

The concentration of drug and the length of treatment used in this study affected the mode of cell death. In the 24 h treatment at higher concentrations of 30 and 10 μg/mL of nordamnacanthal and damnacanthal, the percentage of apoptotic cells was higher as compared to the lower concentrations of 3 and 1 μg/mL. After 72 h, highest percentage of necrotic cells could be seen in the treatment of higher concentrations of both compounds. This outcome is similar to those reported in other forms of cell lines [25,26], suggesting that cells that are not acutely damaged may activate a programmed suicide mechanism while acute cytotoxicity may cause necrosis.

DNA fragmentation and death were prevented in damnacanthal-treated CEM-SS cells by zinc sulphate at 0.1 mM. Zinc was not in itself harmful to the cells, so it appeared that it prevented the activation or action of the endogenous endonuclease that reduces chromosomal DNA to oligonucleosomal fragments [27]. Zinc ion might act on some steps in one or more intervening metabolic processes between the early event of DNA strand breaks and a late consequence of endonucleolytic DNA damage, for instance, by inhibition of the cellular or nuclear uptake calcium, by blocking or competing with calcium for membrane channels or by the inhibition of calmodulin [28]. Zinc may also modify apoptosis by activating protein kinase C or by inhibiting phosphorylases associated with inositol phosphate metabolism [29,30].

On the other hand, zinc failed to interfere with apoptosis stimulated by nordamnacanthal, showing that the optimum concentration of zinc for the prevention of apoptosis varies with different types of stimulus [31]. Even though zinc at the concentrations of approximately 1 to 5 mM has often been ascribed to prevent apoptosis, no attempt has been made to try them in the case of nordamnacanthal. At these concentrations, zinc was found to have damaging effects on CEM-SS and other T-cell acute lymphoblastic leukaemia cells, not the least being the induction of necrosis. It is likely that a cell would need to be essentially intact for the apoptotic death program to be affected. In this case, therefore, general disruption to the biochemical machinery of the cell, and not specific endonuclease inhibition alone, might be responsible for the observed zinc inhibition [32]. It was also possible that zinc modified common process signals for both the change in cell membrane permeability and the earliest DNA cleavage [33] in cells treated with nordamnacanthal. Furthermore, Eron et al. [34] showed that the requirement for high concentrations of extracellular zinc to inhibit apoptosis is probably due to the relatively poor uptake of zinc across the cell membrane [35].

Phosphorylation/dephosphorylation plays a central role in the regulation of cellular proliferation. It seems that the balance of these activities plays an important part in apoptosis, and protein dephosphorylation plays a central role in the process of apoptosis. Okadaic acid is a very potent inhibitor of protein phosphatase 1 (PP1) and protein phosphatase 2A (PP2A), two of the four major protein phosphatases in the cytosol of mammalian cells that dephosphorylate serine and threonine residues, which are involved in regulating a variety of intracellular processes [36]. The mechanism of inhibition is suggested to be the interactions of okadaic acid with the C-terminal domain of PP1 and PP2A [37]. PP1 and PP2A undergo dephosphorylation during apoptosis [38].

Nevertheless, okadaic acid (100 nM) failed to block the occurrence of DNA ladder, or indirectly, apoptosis, induced by nordamnacanthal and damnacanthal, even though the dephosphorylation of PP1 and PP2A was inhibited. Dephosphorylation of these proteins has been speculated to be an important process contributing to apoptosis induced by these two compounds, due to the report that emodin and aloe-emodin, the anthraquinones, for instance, were the inhibitor of protein kinase C [39]. This was based on the fact that PP1 and PP2 have been reported to have the opposite effect to protein kinase C [40]. Nevertheless, studies by Song et al. [41] showed that the failure of okadaic acid to prevent the occurrence of a DNA ladder did not conclude that dephosphorylation of PP1 and PP2A was not involved in the apoptotic cell death by certain drugs. In their experiments, for instance, over the 8 h incubation period, okadaic acid (1 μM) did not affect the viability of CEM-C7 cells, but induced apoptosis after 24 h. In summary, for shorter incubation periods, okadaic acid prevents the morphological changes and DNA fragmentation induced by various stimuli, whereas after longer incubation periods, it is cytotoxic, and the mechanism of cell death appears to be by apoptosis. Since the extent of apoptosis after 24 h was approximately the same in nordamnacanthal- and damnacanthal-treated and untreated cells in the presence of okadaic acid, it seemed that okadaic acid caused apoptosis by one pathway, and yet interfered with cytotoxic nordamnacanthal- and damnacanthal-induced apoptosis, presumably by another pathway.

Cycloheximide (10 μg/mL), the inhibitor of process of translational or protein synthesis, completely prevented apoptosis induced by damnacanthal in CEM-SS cells, but actinomycin D (1 and 10 μg/mL), the transcriptional process or mRNA synthesis inhibitor, failed to prevent the occurrence of a DNA ladder. This result suggests that apoptosis induced by damnacanthal required protein, but not RNA synthesis. The effects of cycloheximide and actinomycin D in protecting cells from death seemed to be different in different systems. Goodall et al. [42] claimed that cycloheximide protective effect was due to the prevention of the expression of proapoptotic genes. Meanwhile, Sánchez-Alcázar et al. [43] stated that cycloheximide prevented the increase of cytochrome c levels in the mitochondrial fraction, the critical step in the apoptotic process. Hoshino et al. [44] showed that cycloheximide and actinomycin D were able to block apoptosis by deactivating caspase-3 activity.

It was also possible that damnacanthal might inactivate protein or proteins suppressing apoptosis, and that cycloheximide prevented apoptosis directly by changing the levels of labile protein or proteins promoting/suppressing apoptosis. However, Chow et al. [45] suggested that cycloheximide just delayed the onset of chromatin degradation without blocking the process of cell death per se. The failure of cycloheximide or actinomycin D to block apoptosis stimulated by nordamnacanthal showed that the event occurs independently of ongoing transcription and protein synthesis. In fact, since treatment with actinomycin D alone induced apoptosis in CEM-SS cells, this showed that the cells already have the apoptotic machinery in place, and the continuous synthesis of a signal is required to suppress spontaneous apoptosis. However, actinomycin D is also regarded as an apoptotic inducing agent by inhibiting RNA synthesis, intercalating in DNA, inducing hypersensitivity of DNA to DNase I and inhibiting both topoisomerase I and II [46].

This suggests that the cells have already expressed the molecular machinery needed to undergo apoptosis upon nordamnacanthal addition, and no further transcription events induced by the compound are needed [47]. It is thus likely that the requirement for de novo protein synthesis is not absolute within a particular cell type, but varies with the inducing stimulus.

Theoretically, new protein synthesis might be required for priming a cell for apoptosis, and for the initiation and execution of the process. The requirement for protein synthesis in damnacanthal-induced apoptosis might be linked to early control events involved with the initiation of the apoptotic process. However, in the case of nordamnacanthal, it seemed likely that the final common effector mechanisms leading to the DNA fragmentation and morphological changes of apoptosis were independent of macromolecular synthesis, allowing the compound that trigger the process down-stream in the activation pathway to bypass the requirement for protein synthesis. Cohen et al. [48] have proposed that the putative suppressor proteins that hold in check the primed apoptotic program within many cells might have a very short half-life, and need to be continuously replenished. Thus, the inhibition of protein synthesis would cause depletion of the short-lived apoptotic suppressor protein, thereby releasing the primed apoptotic death program within the cell. Nordamnacanthal could possibly damage proteins, and trigger apoptosis by directly inactivating the apoptotic suppressor protein, as a result of protein denaturation.

Elevations of the cytosolic Ca^2+^ concentration were involved in the activation of apoptosis by various stimuli and commonly mediated cytotoxicity. Cytosolic Ca^2+^ increases precede endonuclease activation [49]. Damnacanthal from *M. citrifolia* has been reported to increase intracellular Ca^2+^ by releasing Ca^2+^ from internal stores and promoting Ca^2+^ entry in mediating apoptosis [50]. Nevertheless, current results obtained did not support such a view. The presence of the calcium chelator EGTA at concentrations of 0.5, 1 and 8 mM completely failed to prevent nordamnacanthal- and damnacanthal-induced apoptosis in the CEM-SS cells. Moreover, treatment using 8 mM of EGTA per se caused apoptosis. This was probably due to different cell lines being used in these two experiments; human dermal fibroblasts in the former, while acute T-lymphoblastic leukaemia cells in the latter case. An increase in cytosolic Ca^2+^ appeared to be neither induced by nordamnacanthal or damnacanthal, nor be responsible for the induction of apoptosis of the compounds in CEM-SS cells, even though the basal level of cytosolic Ca^2+^ may be required for the activation of an endonuclease.

La Rovere et al. [51] further showed that the induction of apoptosis due to elevations of Ca^2+^ was different for different cell lines. For instance, in the case of childhood acute lymphoblastic leukaemia cells (CEM-C7), the endonuclease that cleaved DNA was not sensitive to calcium, therefore the addition of EGTA gave no preventive effects, unlike in rat thymocytes, where there was a constitutive calcium- and magnesium-dependent endonuclease that promoted internucleosomal DNA cleavage. Gerschenson and Rotello [52] claimed that elevated cytosolic Ca^2+^ is not universally present during induction of apoptosis, and that the activation of endonuclease may be by intracellular acidification rather than an increase in cytosolic Ca^2+^. Therefore, it could be speculated that the elevations of cytosolic Ca^2+^ levels were not critically involved in the triggering of apoptosis in CEM-SS cells by nordamnacanthal and damnacanthal from *M. elliptica*.

The cell cycle analysis indicated that nordamnacanthal and damnacanthal at their IC_50_ values have different mechanisms by which they exert their cytotoxic effects. Nordamnacanthal was found to have cytotoxic effect by inducing apoptosis in CEM-SS cells. Damnacanthal, on the hand, showed a cytostatic effect by causing arrest at the G0/G1 phase of the cell cycle. Arrest at this phase may be due to the inhibition of several tyrosine kinases and LIM-kinase as being shown by damnacanthal from *M. citrifolia* [53,54,55]. This will cause defects or alteration in the receptors, therefore preventing a sufficient amount of a particular growth factor to bind to the cell surface, and an uptake of amino acids, ions and other nutrients, i.e., the criteria needed for a cell to progress further around the cycle, arresting them at G1 phase. This finding showed that the cytotoxic effect (apoptosis) seemed to be independent of the cytostatic effect (block in the cell cycle).

The success of cancer treatment is highly dependent on the diagnosis and prognosis of the cancer. However, the usage of these drugs is often accompanied with undesired side effects such as abdomen pain, mood swings, depression, breathing difficulties, nausea and fatigue [56]. Hematological toxicity, gastrointestinal toxicity ototoxicity, hepatotoxicity, cardiotoxicity and neurotoxicity are also noted following the use of chemotherapeutic agents [57,58,59,60,61]. If the treatments with damnacanthal and nordamnacanthal have fewer health consequences than the drugs currently in use in the clinical setting, they have high potential to be established as anticancer drugs. However, further tests in vivo and clinical research are required to know the possible effects exhibited by these anthraquinones.

## 4. Materials and Methods

### 4.1. Materials

#### 4.1.1. Cells and Compounds

The T-lymphoblastic leukaemia cell line, CEM-SS, (anchorage-dependent and suspension cells) were obtained from the American Type Culture Collection (ATCC), the National Cancer Institute (NCI) and the RIKEN Cell Bank (RCB).

#### 4.1.2. Compounds

*Morinda elliptica* were collected from Port Dickson, Negeri Sembilan and identified by Mr Anthonysamy Sivarimuthu of the Department of Biology. A voucher specimen (Voucher number SK2391/14) was deposited in the herbarium of Institute of Bioscience, Universiti Putra Malaysia, Serdang. *Morinda elliptica* roots were used to isolate nordamnacanthal and damnacanthal, as detailed in Ismail et al. [62]. *Morinda elliptica*’s roots were washed under running tap water, air dried (24–26 °C), chopped up and ground. Then, 12 kg of the powder was soaked at room temperature (24–26 °C) in dichloromethane (CH_2_Cl_2_), at an amount that entirely immerses the powdered root for 36 h.

Fresh CH_2_Cl_2_ was added after the earlier solvent was filtered out, and this process was repeated for a total of three times. The combined filtrate was evaporated under reduced pressure to give a brown colored residue of 142 g. Then, 26 g of the crude extract was dissolved in CHCl_3_, and absorbed a packed column (10 cm × 50 cm) onto an acid-washed silica gel, which was earlier shaken with 4% oxalic acid for 30 min, filtered and dried. Ninety-five fractions were collected and combined (based on TLC pattern) into six major quinone-containing fractions (labeled A, B, C, D, E and F) for further separation procedures. After column chromatography, fraction A was re-chromatographed using a smaller column (300 mm × 35 mm) packed with 2% acid washed silica gel and eluted with a mixture of CH_2_Cl_2_ and petrol. Nordamnacanthal was found to be the major component of fraction B and C, separated by TLC developed in CHCl_3_. An amount of 1.02 g nordamnacanthal (with a melting point of 214–218 °C) was isolated after recovery of the major orange band. The yellow band that appeared after the major orange band of fraction B was then separated using chromatotron eluted with CHCl_3_ to produce 196.4 g of damnacanthal. The spectroscopic values of nordamnacanthal and damnacanthal were verified with literature values [63,64].

The powdered-form of nordamnacanthal and damnacanthal were dissolved in dimethylsulphoxide (DMSO, Sigma, St. Louis, MO, USA). The percentage of DMSO used to dissolve the compound were ensured to be lesser than 5%. The solution was prepared with a serum-free culture medium, RPMI 1640 (Sigma-Aldrich, St. Louis, MO, USA) and stored at 4 °C.

### 4.2. Methods

#### 4.2.1. Cell Lines

CEM-SS cells were grown in RPMI 1640 medium (Sigma, St. Louis, MO, USA). The medium was supplemented with 10% foetal calf serum (Sera Lab, Sussex, UK) and antibiotics (100 units/mL penicillin and 100 µg/mL streptomycin) (Sigma, St. Louis, MO, USA), and incubated at 37 °C under 5% CO2 in a humidified atmosphere. The cells were ensured to be having exponential growth before any procedure on the cells was carried out.

#### 4.2.2. Cytotoxicity Assay

A volume of 100 µL of nordamnacanthal and damnacanthal at concentrations of 30, 10, 3, 1, 0.3, 0.1 and 0.03 µg/mL were added to CEM-SS cell suspension in complete growth media at 1 × 10^5^ cells/mL. Controls that contained only the cells were included for each sample. The assay for each concentration of the compound was performed in triplicate. The plate was then incubated at 37 °C, 5% CO_2_, 90% humidity for 72 h.

3-[4,5-dimethylthizol-2-yl]-2,5-diphenyltetrazolium bromide (MTT, Sigma, St. Louis, MO, USA) assay was carried out as reported by Mosmann [65]. The MTT solution was added directly into wells containing cells, complete growth medium with or without the tested compound. The plate was then incubated for 4 h at 37 °C to allow MTT metabolism to formazan. Subsequently, the supernatant was aspirated and 100 µl of acid-isopropanol (0.04 M HCl in propan-2-ol) was added and mixed thoroughly to dissolve the dark blue formazan crystals. The optical density (OD) was measured on an automated spectrophotometric EL 340 multiplate/microelisa reader (Bio-Tek Instruments Inc., Winooski, VT, USA) using test and reference wavelength of 570 and 630 nm, respectively. The cytotoxic dose that killed cells by 50% (IC_50_) was determined from absorbance (OD) versus concentration curve.

#### 4.2.3. DNA Fragmentation Assay

CEM-SS cells with a density of 1 × 10^5^ cells/mL were treated with 30, 10, 3 and 1 µg/mL of nordamnacanthal and damnacanthal. Controls that contained only the cells were included for each sample. Sampling was done in every 8 h for 3 days (72 h). The cells were also treated with a combination of 30 µg/mL nordamnacanthal or damnacanthal and 10 µg/mL cycloheximide (Sigma, USA)/0.1 mM zinc sulphate (Sigma, USA)/1 µg/mL actinomycin D (Sigma, USA)/500 nM okadaic acid (Sigma, USA)/1 or 8 mM EGTA (Sigma, USA) and incubated for 24 h.

Cells were harvested and lysed with lysis buffer containing 150 mM NaCl, 100 mM Tris pH 8.0–100 mM EDTA and 0.5% sodium dodecylsulfate in the presence of proteinase K (0.25 mg/mL) (Promega) at 50 °C for 1 h. Then, 0.1 mg/mL RNase A (Promega) solution was added, and the incubation continued at 50 °C for another hour. The total genomic DNA was then isolated with phenol/chloroform/isoamyl alcohol (25:24:1). Two volumes of cold ethanol were added and mixed thoroughly. The DNA was precipitated by centrifugation at 2500 rpm (1250× *g*) for 5 min. After being air-dried, the DNA was dissolved in TE buffer (10 mM Tris-HCl, pH 7.5–10 mM EDTA) overnight at 4 °C. The amount of TE depends on the amount of DNA spooled (normally 50–200 µL). DNA was electrophoresed in 1% agarose gel, stained with ethidium bromide and was visualized by UV illumination (245 nm) using polaroid 667 and a red filter. The DNA standard used for size comparison was HindIII digests of lambda phage DNA.

#### 4.2.4. Working Procedure for the ELISA

Cells with a density of 1 × 10^5^ cells/mL were treated with 30 µg/mL of nordamnacanthal and damnacanthal, and incubated for 24, 48 and 72 h at 37 °C and 5% CO_2_. Control wells of untreated cell population were also included. Culture medium without the compound was used as a negative control. The cell pellet was then resuspended/lysed in 200 µl lysis buffer and incubated for 30 min at room temperature (=lysis). The lysate was subsequently centrifuged at 1000 rpm (200× *g*) for 10 min.

Twenty microlitres of cell lysate were transferred into the MTP. Eighty microlitres of the immunoreagent-mix was added to each well. The MTP was covered with an adhesive cover foil and incubated with gentle shaking (500 rpm) for 2 h at room temperature. The content was discarded and rinsed three times, before 100 µL of substrate solution was added into each well of the MTP-modules and incubated on a plate shaker at 250 rpm until the color developed. The OD was measured on an automated spectrophotometric EL 340 multiplate/microelisa reader (Bio-Tek Instruments Inc.) against substrate solution as a blank using test and reference wavelength of 405 nm and 492 nm, respectively. The specific enrichment of mono- and oligonucleosomes released into the cytoplasm for these values were calculated using the following formula,

$$\begin{array}{c} \mathrm{Enrichment}\;\mathrm{factor}=\frac{\mathrm{mUof}\;\mathrm{sample}\;\mathrm{dying}\;\mathrm{or}\;\mathrm{dead}\;\mathrm{cells}}{\mathrm{mU}\;\mathrm{of}\;\mathrm{corresponding}\;\mathrm{cells}\;\mathrm{without}\;\mathrm{treatment}}\\ \mathrm{mU}=\mathrm{absorbance}\;\left[10^{-3}\right] \end{array}$$

#### 4.2.5. Flow Cytometric Analyses

CEM-SS cells with a density of 1 × 10^5^ cells/mL were treated with nordamnacanthal and damnacanthal at the IC_50_, for 24 h and 48 h. Controls that contained only the cells were included for each sample. The cells were harvested washed twice with PBS and fixed for flow cytometry analyses. Briefly, cell pellets were fixed with 50% cold ethanol (0.5 mL PBS + 0.5 mL absolute ethanol) at 4 °C for 15 min. The ethanol was discarded before the cell pellet was then resuspended in 0.425 mL of PBS, 25 μL of propidium iodide (1 mg/mL), and 50 μL of RNAse A (1 mg/mL) for 20 min. The cell cycle kinetics were analyzed using FACScan and CELLFIT software (Becton Dickinson, San Jose, CA, USA).

## 5. Conclusions

The human CEM-SS cell line showed the highest sensitivity towards nordamnacanthal, with the IC_50_ of 1.7 μg/mL. Damnacanthal was less toxic to this cell line, with the IC_50_ of 10 μg/mL. The programmed cell death was then proven at the molecular level by the formation of a distinctive ladder-like pattern on the agarose gel, as a result of cleavage of the chromatin at the internucleosomal regions generating fragments that were multiples of 200 bp. The DNA fragmentation is due to the activation of the Mg^2+^/Ca^2+^-dependent endonuclease. The induction of apoptosis by nordamnacanthal was different from the one induced by damnacanthal, in a way that it occurs independently of ongoing transcription process. Nevertheless, in both cases, the process of dephosphorylation of protein phosphates 1 and 2A, the ongoing protein synthesis and the elevations of the cytosolic Ca^2+^ concentration were not needed for apoptosis to take place. Nordamnacanthal was found to have a cytotoxic effect by inducing apoptosis in CEM-SS cells. Damnacanthal, on the other hand, showed a cytostatic effect by causing arrest at the G0/G1 phase of the cell cycle. This finding showed that the cytotoxic effect (apoptosis) seemed to be independent of the cytostatic effect (block in the cell cycle).

## Figures and Tables

**Table 1:** Summary of cell cycle distribution of CEM-SS cells after 24 h and 48 h incubation with nordamnacanthal and damnacanthal at their respective IC_50_ values.

	Time after Treatment		
	Control (0 h)	24 h	48 h
**Nordamnacanthal**			
Sub-G1 (%)	2.83	32.54	41.15
G0/G1 (%)	31.77	4.72	12.24
S (%)	45.96	59.36	40.19
G2/M (%)	19.43	3.24	6.41
**Damnacanthal**			
Sub-G1 (%)	2.83	4.07	6.12
G0/G1 (%)	31.77	63.31	70.41
S (%)	45.96	20.62	11.87
G2/M (%)	19.43	11.23	10.78

## Data Availability

Data is contained within the article.

## Sample Availability

Samples of the compounds damnacanthal and nordamnacanthal are available from the authors.

## References

[1] GLOBOCAN (2018). World. https://gco.iarc.fr/today/data/factsheets/populations/900-world-fact-sheets.pdf.

[2] Bray F., Ferlay J., Soerjomataram I., Siegel R.L., Torre L.A., Jemal A. (2018). Global cancer statistics 2018: GLOBOCAN estimates of incidence and mortality worldwide for 36 cancers in 185 countries. CA Cancer J. Clin..

[3] Greaves M.A. (2018). Causal mechanism for childhood acute lymphoblastic leukaemia. Nat. Rev. Cancer.

[4] Samra B., Jabbour E., Ravandi F., Kantarjian H., Short N.J. (2020). Evolving therapy of adult acute lymphoblastic leukemia: State-of-the-art treatment and future directions. J. Hematol. Oncol..

[5] Alias H., Doris Lau S.C., Loh C.K., Harrison C.J., Eswaran J. (2019). Improved Treatment of Childhood ALL in Malaysia. Blood.

[6] Burkill I.H. (1966). A Dictionary of the Economic Products of the Malay Peninsula.

[7] Jasril L.N.H., Lajis N.H., Abdullah M.A., Sukari M.A., Ali A.M. (2003). Antitumor promoting and antioxidant activities of anthraquinones isolated from cell suspension culture of Morinda elliptica. Asia Pac. J. Mol. Biol. Biotechnol..

[8] Ali A.M., Ismail N.H., Mackeen M.M., Yazan L.S., Mohamed S.M., Ho A.S.H., Lajis N.H. (2000). Antiviral, cytotoxic and antimicrobial activites of anthraquinones isolated from the roots of *Morinda elliptica*. Pharm. Biol..

[9] Akhtar M.N., Zareen S., Yeap S.K., Ho W.Y., Lo K.M., Hasan A., Alitheen N.B. (2013). Total Synthesis, Cytotoxic Effects of Damnacanthal, Nordamnacanthal and Related Anthraquinone Analogues. Molecules.

[10] Subramani T., Yeap S.K., Ho W.Y., Ho C.L., Osman C.P., Ismail N.H., Rahman N.M., Alitheen N.B. (2015). Nordamnacanthal potentiates the cytotoxic effects of tamoxifen in human breast cancer cells. Oncol. Lett..

[11] Abu N., Zamberi N.R., Yeap S.K., Nordin N., Mohamad N.E., Romli M.F., Rasol N.E., Subramani T., Ismail N.H., Alitheen N.B. (2018). Subchronic toxicity, immunoregulation and anti-breast tumor effect of Nordamnacantal, an anthraquinone extracted from the stems of *Morinda citrifolia* L.. BMC Complement. Altern. Med..

[12] Lv L., Chen H., Ho C.T., Sang S. (2011). Chemical components of the roots of Noni (*Morinda citrifolia*) and their cytotoxic effects. Fitoterapia.

[13] Nualsanit T., Rojanapanthu P., Gritsanapan W., Lee S.H., Lawson D., Baek S.J. (2012). Damnacanthal, a noni component, exhibits antitumorigenic activity in human colorectal cancer cells. J. Nutr. Biochem..

[14] Shaghayegh G., Alabsi A.M., Ali-Saeed R., Ali A.M., Vincent-Chong V.K., Ismail N.H., Choon Y.F., Zain R.B. (2017). Effects of Damnacanthal and Nordamnacanthal on Proliferation, Apoptosis, and Migration of Oral Squamous Cell Carcinoma Cells. Asian Pac. J. Cancer Prev..

[15] García-Vilas J.A., Quesadal A.R., Medina M.A. (2015). Damnacanthal, a noni anthraquinone, inhibits c-Met and is a potent antitumor compound against Hep G2 human hepatocellular carcinoma cells. Sci. Rep..

[16] Ismail N., Mohamad H., Mohidin A., Lajis N.H. (2002). Antioxidant activity of anthraquinones from *Morinda elliptica*. Nat. Prod. Sci..

[17] Shami A.M.M. (2018). Antibacterial and antioxidant properties of anthraquinones fractions from *Morinda citrifolia* fruit. J. Rep. Pharm. Sci..

[18] Kamei H., Koide T., Kojima T., Hashimoto Y., Hasegawa M. (1998). Inhibition of cell growth in culture by quinones. Cancer Biother. Radiopharm..

[19] Kamil M., Haque E., Mir S.S., Irfan S., Hasan A., Sheikh S., Alam A., Ansari K.M., Nazir A. (2019). Hydroxyl Group Difference between Anthraquinone Derivatives Regulate Different Cell Death Pathways via Nucleo-Cytoplasmic Shuttling of p53. Anticancer Agents Med. Chem..

[20] Li Y., Jiang J.G. (2018). Health functions and structure-activity relationships of natural anthraquinones from plants. Food Funct..

[21] Sachet M., Liang Y.Y., Oehler R. (2017). The immune response to secondary necrotic cells. Apoptosis.

[22] Saadat R.Y., Saeidi N., Zununi Vahed S., Barzegari A., Barar J. (2015). An update to DNA ladder assay for apoptosis detection. Bioimpacts.

[23] Shaghayegh G., Alabsi A.M., Ali-Saeed R., Ali A.M., Vincent-Chong V.K., Zain R.B. (2016). Cell cycle arrest and mechanism of apoptosis induction in H400 oral cancer cells in response to Damnacanthal and Nordamnacanthal isolated from *Morinda citrifolia*. Cytotechnology.

[24] Walker P.R., Kokileva L., LeBlanc J., Sikorska M. (1993). Detection of the intial stages of DNA fragmentation in apoptosis. Biotechniques.

[25] Hao L., Zhao Y., Li Z., He H., Liang Q., Zhang Z., Shi Z., Zhang P., Han C. (2017). Tumor necrosis factor-related apoptosis-inducing ligand inhibits proliferation and induces apoptosis of prostate and bladder cancer cells. Oncol. Lett..

[26] Xiao Q., Lu Y., Chen X. (2020). Oleanolic Acid Induces Apoptosis and Necrosis in LO2 Cells. Indian J. Pharm. Sci..

[27] Duvall E., Wyllie A.H. (1986). Death and the cell. Immunol. Today.

[28] Kumar V., Chichili V.P., Tang X., Sivaraman J. (2013). A novel trans conformation of ligand-free calmodulin. PLoS ONE.

[29] Waring P., Kos F.J., Mullbacher A. (1991). Apoptosis or programmed cell death. Med. Res. Rev..

[30] Marini M., Musiani D. (1998). Micromolar Zinc Affects Endonucleolytic Activity in Hydrogen Peroxide-Mediated Apoptosis. Exp. Cell Res..

[31] Gammoh N.Z., Rink L. (2017). Zinc in infection and inflammation. Nutrients.

[32] Harmon B.V., Forster T.H., Collins R.J., Lavin M., Watters D. (1993). Hyperthermia-Induced apoptosis. Programmed Cell Death: The Cellular and Molecular Biology of Apoptosis.

[33] Bicknell G.R., Snowden R.T., Cohen G.M. (1994). Formation of high molecular mass DNA fragments is a marker of apoptosis in the human leukaemic cell line, U397. J. Cell Sci..

[34] Eron S.J., MacPherson D.J., Dagbay K.B., Hardy J.A. (2018). Multiple Mechanisms of Zinc-Mediated Inhibition for the Apoptotic Caspases-3, -6, -7, and -8. ACS Chem. Biol..

[35] Radford R.J., Chyan W., Lippard S.J. (2013). Peptide-Based Targeting of Fluorescent Zinc Sensors to the Plasma Membrane of Live Cells. Chem. Sci..

[36] Haneji T., Hirashima K., Teramachi J., Morimoto H. (2013). Okadaic acid activates the PKR pathway and induces apoptosis through PKR stimulation in MG63 osteoblast-like cells. Int. J. Oncol..

[37] Takai A., Eto M., Hirano K., Takeya K., Wakimoto T., Watanabe M. (2018). Protein phosphatases 1 and 2A and their naturally occurring inhibitors: Current topics in smooth muscle physiology and chemical biology. J. Physiol. Sci..

[38] Dedinszki D., Kiss A., Márkász L., Márton A., Tóth E., Székely L., Erdődi F. (2015). Inhibition of protein phosphatase-1 and -2A decreases the chemosensitivity of leukemic cells to chemotherapeutic drugs. Cell. Signal..

[39] Janeczko M., Masłyk M., Kubiński K., Golczyk H. (2017). Emodin, a natural inhibitor of protein kinase CK2, suppresses growth, hyphal development, and biofilm formation of Candida albicans. Yeast.

[40] Nishizuka Y. (1988). The molecular heterogenity of protein kinase C and its implications for cellular regulation. Nature.

[41] Song Q., Baxter G.D., Kovacs E.M., Findik D., Lavin M.F. (1992). Inhibition of apoptosis in human tumour cells by okadaic acid. J. Cell. Physiol..

[42] Goodall K.J., Finch-Edmondson M.L., van Vuuren J., Yeoh G.C., Gentle I.E., Vince J.E., Ekert P.G., Vaux D.L., Callus B.A. (2016). Cycloheximide Can Induce Bax/Bak Dependent Myeloid Cell Death Independently of Multiple BH3-Only Proteins. PLoS ONE.

[43] Sánchez-Alcázar J.A., Khodjakov A., Schneider E. (2001). Anticancer Drugs Induce Increased Mitochondrial Cytochrome C Expression That Precedes Cell Death. Cancer Res..

[44] Hoshino M., Qi M.L., Yoshimura N., Miyashita T., Tagawa K., Wada Y., Enokido Y., Marubuchi S., Harjes P., Arai N. (2006). Transcriptional repression induces a slowly progressive atypical neuronal death associated with changes of YAP isoforms and p73. J. Cell Biol..

[45] Chow S.C., Peters I., Orrenius S. (1995). Reevaluation of the role of de novo protein synthesis in rat thymocyte apoptosis. Exp. Cell Res..

[46] Bensaude O. (2011). Inhibiting eukaryotic transcription. Which compound to choose? How to evaluate its activity?. Transcription.

[47] Choi J.W., Jung S.E. (1999). Lovastatin-Induced proliferation inhibition and apoptosis in C6 glial cells. J. Pharmacol. Exp. Ther..

[48] Cohen J.J., Duke R.C., Fadok V.A., Sellins K.S. (1992). Apoptosis and programmed cell death in immunity. Annu. Rev. Immunol..

[49] Rizzuto R., Pinton P., Ferrari D., Chami M., Szabadkai G., Magalhães P.J., Virgilio F.D., Pozzan T. (2003). Calcium and apoptosis: Facts and hypotheses. Oncogene.

[50] Aoki K., Parent A., Zhang J. (2000). Mechanism of damnacanthal-induced [Ca2+]i elevation in human dermal fibroblasts. Eur. J. Pharmacol..

[51] La Rovere R.M.L., Roest G., Bultynck G., Parys J.B. (2016). Intracellular Ca^2+^ signaling and Ca^2+^ microdomains in the control of cell survival, apoptosis and autophagy. Cell Calcium.

[52] Gerschenson L.E., Rotello R.J. (1992). Apoptosis: A different type of cell death. FASEB J..

[53] Dibwe D.F., Awale S., Kadota S., Tezuka Y. (2012). Damnacanthal from the Congolese medicinal plant Garcinia huillensis has a potent preferential cytotoxicity against human pancreatic cancer PANC-1 cells. Phytother. Res..

[54] Ohashi K., Sampei K., Nakagawa M., Uchiumi N., Amanuma T., Aiba S., Oikawa M., Mizuno K. (2014). Damnacanthal, an effective inhibitor of LIM-kinase, inhibits cell migration and invasion. Mol. Biol. Cell.

[55] García-Vilas J.A., Pino-Ángeles A., Martínez-Poveda B., Quesada A.R., Medina M.Á. (2017). The noni anthraquinone damnacanthal is a multi-kinase inhibitor with potent anti-angiogenic effects. Cancer Lett..

[56] Jim H.S., Jacobsen P.B., Phillips K.M., Wenham R.M., Roberts W., Small B.J. (2013). Lagged relationships among sleep disturbance, fatigue, and depressed mood during chemotherapy. Health Psychol..

[57] Ansari L., Shiehzadeh F., Taherzadeh Z., Nikoofal-Sahlabadi S., Momtazi-borojeni A.A., Sahebkar A., Eslami S. (2017). The most prevalent side effects of pegylated liposomal doxorubicin monotherapy in women with metastatic breast cancer: A systematic review of clinical trials. Cancer Gene Ther..

[58] Dar W., Hussain M., Aziz S.A., Mohammad G., Wani B., Latief M. (2017). Uncommon Adverse Effects of Commonly Used Chemotherapeutic Agents in Medical Oncology Practice: A Series of Two Cases of Hand-Foot Syndrome. Indian J. Med. Paediatr. Oncol..

[59] Jameel P.Z., Lohiya S., Dongre A., Damke S., Lakhkar B.B. (2020). Concurrent diabetic ketoacidosis and pancreatitis in Paediatric acute lymphoblastic leukemia receiving L-asparaginase. BMC Pediatr..

[60] Qi L., Luo Q., Zhang Y., Jia F., Zhao Y., Wang F. (2019). Advances in Toxicological Research of the Anticancer Drug Cisplatin. Chem. Res. Toxicol..

[61] Rios A., Cen P., Dinh B., Mays S.R., Patel A.B. (2019). Dramatic response of nivolumab-associated psoriasiform dermatitis to etoposide. Eur. J. Cancer.

[62] Ismail N.H., Ali A.M., Aimi N., Kitajima M., Takayama H., Lajis N.H. (1997). Anthraquinones from *Morinda elliptica*. Phytochemistry.

[63] Hirose Y. (1960). Syntheses of Damnacanthal, Damnacanthol, Norjuzunal and Norjuzunol, the Coloring Matters of Damnacanthus spp.. Chem. Pharm. Bull..

[64] Adnan N.E. (2018). Isolation and photophysical properties of Di- and Tri-substituted natural anthraquinones from Malaysian *Morinda citrifolia*. Sains Malays..

[65] Mossman T. (1983). Rapid colorimetric assay for cellular growth and survival: Application to proliferation and cytotoxicity assays. J. Immunol. Methods.

